# Meta-analysis assessing the effectiveness of SGLT2i+GLP1RA combination therapy versus monotherapy on cardiovascular and cerebrovascular outcomes in diabetic patients

**DOI:** 10.3389/fphys.2022.1028486

**Published:** 2022-11-07

**Authors:** Lixin Du, Jiao Qin, Dengchuan Wang, Yunhui Zhao, Ning Xu, Chaowen Wu, Jianpeng Yuan

**Affiliations:** ^1^ Department of Medical Imaging, Shenzhen Longhua District Central Hospital, Shenzhen, China; ^2^ Office of Medical Ethics, Shenzhen Longhua District Central Hospital, Shenzhen, China; ^3^ Department of Endocrinology, Shenzhen Longhua District Central Hospital, Shenzhen, China; ^4^ Department of Radiology, The Seventh Affiliated Hospital, Sun Yat-sen University, Shenzhen, China

**Keywords:** SGLT2I, glp1 agonists, myocardial infarction, stroke, hospitalization for heart failure, mortality

## Abstract

Relevant meta-analyses have confirmed the cardiovascular and renal benefits of sodium-glucose cotransporter 2 inhibitors (SGLT2i) and glucagon-like peptide-1 receptor agonists (GLP1RA) among patients with type 2 diabetes (T2D) and/or cardiorenal disease. However, it is not established whether the combination therapy of SGLT2i and GLP1RA will yield an additive benefit on cardiorenal endpoints. Lopez and colleagues recently did a cohort study (Lopez et al., Am. J. Cardiol., 2022, 181, 87–93) and aimed to address this issue. However, their findings are not consistent with those of previous studies. To confirm Lopez et al.’s findings (Lopez et al., Am. J. Cardiol., 2022, 181, 87–93) and address the aforementioned inconsistencies, we conducted a meta-analysis based on relevant studies. Our meta-analysis identified that SGLT2i + GLP1RA combination therapy was significantly associated with the reduced risks of cardiovascular/cerebrovascular atherosclerotic, heart failure-associated, and death outcomes compared with SGLT2i/GLP1RA monotherapy. These might support this combination therapy used for better reducing cardiovascular and death events in T2D patients, especially in those with high or very high cardiovascular risk. This is a commentary on a previous article (Lopez et al.’s study (Lopez et al., Am. J. Cardiol., 2022, 181, 87–93)) published outside of Frontiers. Therefore, we submitted this manuscript as an Opinion article, as suggested in the Author Guidelines.

## Introduction

Increasing meta-analyses (Sattar et al., 2021d; McGuire et al., 2021; Ali et al., 2022) based on the cardiovascular outcome trials (CVOTs) of sodium-glucose cotransporter 2 inhibitors (SGLT2i) and glucagon-like peptide-1 receptor agonists (GLP1RA) have confirmed the cardiovascular and renal benefits of these two drug classes among patients with type 2 diabetes (T2D) and/or cardiorenal disease. Hence, SGLT2i and GLP1RA have been recommended in T2D patients and patients with heart failure or chronic kidney disease, to prevent cardiorenal and death endpoints by several clinical guidelines (Buse et al., 2019; McDonagh et al., 2021; Mancini et al., 2022). However, it is not established whether the combination therapy of SGLT2i and GLP1RA will yield an additive benefit on these endpoints.

Recently, Lopez and colleagues did a meaningful cohort study (Lopez et al., 2022) focusing on the cardiovascular effectiveness of the combination therapy of SGLT2i and GLP1RA *versus* SGLT2i monotherapy in patients with T2D. Accordingly, the authors identified that SGLT2i + GLP1RA combination therapy was associated with the lower risks of the composite cardiovascular outcome [defined as a composite of myocardial infarction, stroke, or all-cause mortality (ACM)] and ACM but with the similar risk of hospitalization for heart failure (HHF) compared with SGLT2i monotherapy. These findings are interesting and clinically relevant. However, these findings are not consistent with those of previous studies. For example, Lam et al. (Lam et al., 2022) found that the combination therapy *versus* SGLT2i monotherapy was associated with the similar risk of the composite cardiovascular outcome [i.e., major adverse cardiovascular events (MACE), defined as a composite of myocardial infarction, stroke, or cardiovascular mortality (CVM)] [hazard ratio (HR) 0.70, 95% confidence interval (CI) 0.37–1.30] but with the lower risk of HHF (HR 0.23, 95% CI 0.05–0.97). Moreover, Dave et al. (Dave et al., 2021) observed the similar risk of ACM (HR 0.68, 95% CI 0.40–1.14) between the combination therapy and GLP1RA monotherapy. To confirm Lopez et al.’s findings (Lopez et al., 2022) and address the aforementioned inconsistencies, we conducted a meta-analysis based on those studies reporting the effectiveness of SGLT2i + GLP1RA combination therapy *versus* SGLT2i or GLP1RA monotherapy on cardiovascular and cerebrovascular outcomes in T2D patients.

## Methods

This meta-analysis was done according to the PRISMA statement (Moher et al., 2009). We searched Embase, Web of science, and PubMed from inception date to August 2022 using the following search strategies (showing PubMed strategies as an example): [“Diabetes Mellitus, Type 2” [Mesh] OR “diabetes” (all fields)] AND (Sodium-Glucose Transporter 2 Inhibitors (MH) OR “Sodium glucose transporter 2 inhibitor*” (TIAB) OR “Sodium glucose cotransporter 2 inhibitor*” (TIAB) OR “Sodium glucose co-transporter 2 inhibitor*” (TIAB) OR SGLT*(TIAB) OR Gliflozin*(tiab) OR “Empagliflozin” (tiab) OR “Dapagliflozin” (tiab) OR “Canagliflozin” (tiab) OR “ertugliflozin” (tiab) OR “sotagliflozin” (tiab)] AND [“glucagon-like peptide 1 receptor agonist*” (TIAB) OR “GLP1*” (TIAB) OR lixisenatide (TIAB) OR liraglutide (TIAB) OR semaglutide (TIAB) OR exenatide (TIAB) OR albiglutide (TIAB) OR dulaglutide (TIAB) OR Efpeglenatide (TIAB)] AND [“cardiovascular” (tiab) OR “cardiac” (tiab) OR “heart failure” (tiab) OR “myocardial infarction” (TIAB) OR stroke (tiab) OR “MACE” (tiab) OR “death” (tiab) OR “mortality” (tiab)]. Studies eligible to be included were studies reporting the effectiveness of SGLT2i + GLP1RA combination therapy *versus* SGLT2i or GLP1RA monotherapy on cardiovascular outcomes in T2D patients. To our knowledge, in the studies reporting the subgroup/secondary/post hoc analyses of randomized CVOTs of SGLT2i and GLP1RA, participants in the combination therapy group and participants in the monotherapy group did not derive from random allocation any longer. Therefore, these studies should not be considered as randomized trials, but as observational studies. Accordingly, we considered these studies as well as real observational studies in this meta-analysis. Three outcomes of interest were MACE, a composite of CVM or HHF, and ACM. When MACE was not available, a composite of myocardial infarction, stroke, or ACM was used instead. When a composite of CVM/HHF was not available, HHF was used instead. Meta-analyses were done based on the HRs and 95% CIs extracted from included studies and using the random-effects restricted maximum likelihood model. We performed subgroup analyses according to type of monotherapy (SGLT2i or GLP1RA), and calculated *p*-value for subgroup difference (*P*_*subgroup*_) using Cochran’s Q test. We detected publication bias using Egger tests and funnel plots. We performed all the statistical analyses in Stata/MP (version 16.0).

## Results

We included a total of nine studies (Clegg et al., 2019; Arnott et al., 2020; Cannon et al., 2020; Bhatt et al., 2021a; Bhatt et al., 2021b; Cahn et al., 2021; Dave et al., 2021; Lam et al., 2022; Lopez et al., 2022) in this meta-analysis. Compared to SGLT2i/GLP1RA monotherapy, SGLT2i + GLP1RA combination therapy was associated with a 30% reduction in risk of MACE (HR 0.70, 95% CI 0.54–0.91; Figure 1.1), a 31% reduction in risk of CVM/HHF (HR 0.69, 95% CI 0.50–0.95; Figure 1.2), and a 57% reduction in risk of ACM (HR 0.43, 95% CI 0.24–0.76; Figure 1.3). Subgroup analyses results showed that type of control (i.e., type of monotherapy) did not significantly affect the relative effectiveness of SGLT2i + GLP1RA combination therapy *versus* monotherapy on MACE (*P*_*subgroup*_ = 0.14; Supplementary Figure S1) and CVM/HHF (*P*_*subgroup*_ = 0.86; Supplementary Figure S2). Although type of control significantly affected the relative effectiveness of SGLT2i + GLP1RA combination therapy *versus* monotherapy on ACM (*P*_*subgroup*_ = 0.03; Supplementary Figure S3), the combination therapy was significantly associated with the lower risk of ACM whether compared with GLP1RA (HR 0.59, 95% CI 0.38–0.93) or compared with SGLT2i (HR 0.28, 95% CI 0.17–0.46). Funnel plots and Egger tests (Figures 1.4–1.6) did not reveal any publication bias as for these three outcomes (*P*_*Egger*_: 0.908, 0.972, and 0.953, respectively).

**FIGURE 1:**
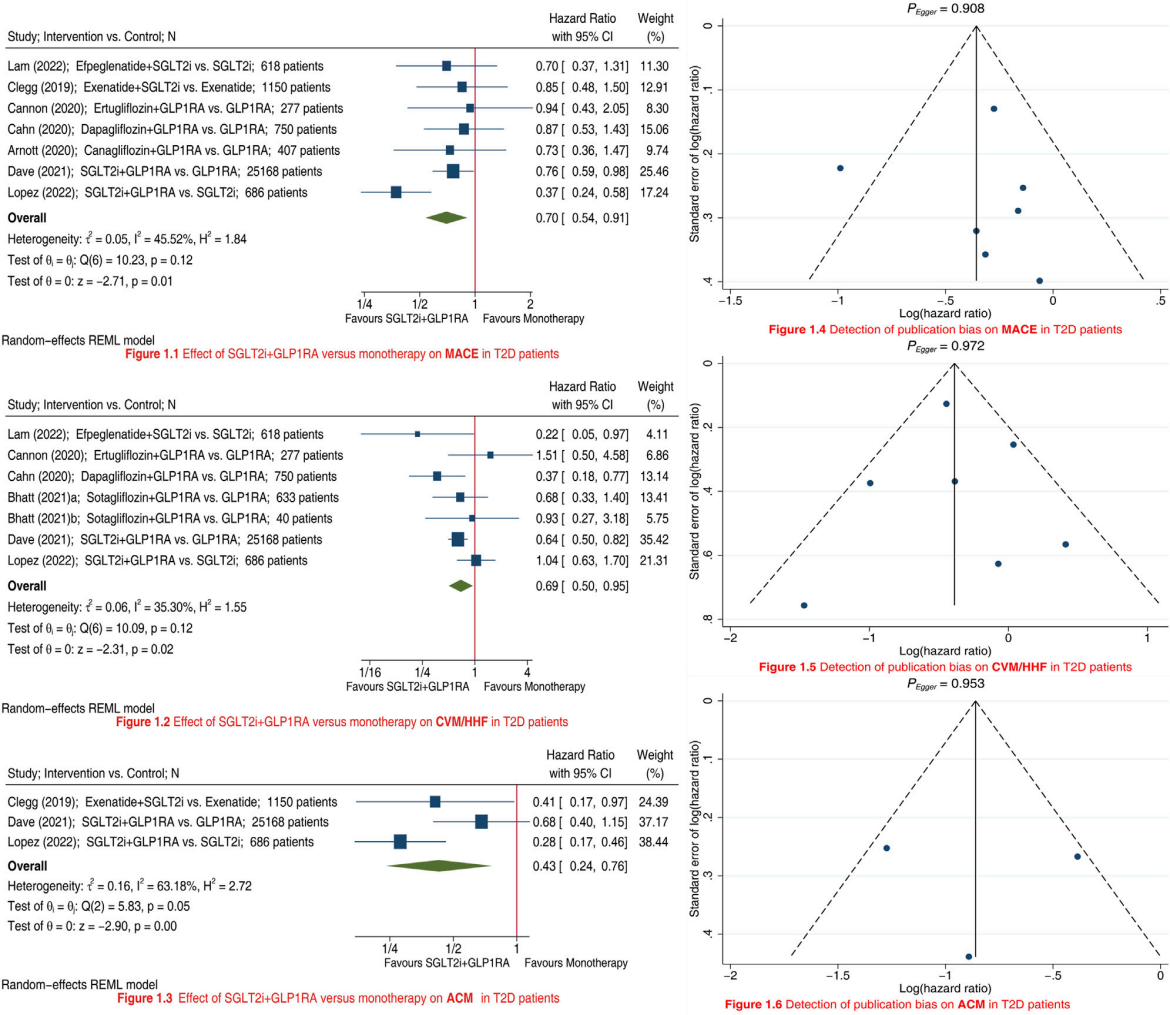
Meta-analyses showing the effects of SGLT2i + GLP1RA combination therapy *versus* SGLT2i/GLP1RA monotherapy on MACE (Panel 1.1), CVM/HHF (Panel 1.2), and ACM (Panel 1.3) in T2D patients; and detection of publication bias on MACE (Panel 1.4), CVM/HHF (Panel 1.5), and ACM (Panel 1.6). SGLT2i = sodium-glucose cotransporter 2 inhibitors. GLP1RA = glucagon-like peptide-1 receptor agonists. MACE = major adverse cardiovascular events. CVM = cardiovascular mortality. HHF = hospitalization for heart failure. ACM = all-cause mortality. T2D = type 2 diabetes. CI = confidence interval.

## Discussion

Previous meta-analyses (Castellana et al., 2019; Mantsiou et al., 2020; Singh and Singh, 2022) revealed that compared with SGLT2i/GLP1RA monotherapy SGLT2i + GLP1RA combination therapy conferred a greater reduction in HbA1c, body weight, and systolic blood pressure; but did not assess or did not have a sufficient power to assess relevant clinical endpoints such as MACE and ACM. On the contrary, our meta-analysis is the first one that focused on addressing the relative effectiveness of SGLT2i + GLP1RA combination therapy *versus* SGLT2i/GLP1RA monotherapy on cardiovascular/cerebrovascular and death endpoints in T2D patients. Our meta-analysis identified that SGLT2i + GLP1RA combination therapy was significantly associated with reduced risks of MACE, CVM/HHF, and ACM compared with SGLT2i/GLP1RA monotherapy. These findings confirmed Lopez et al.‘s findings (Lopez et al., 2022) regarding to the composite cardiovascular outcome and ACM, and updated Lopez et al.‘s findings (Lopez et al., 2022) regarding to HHF. These might support this combination therapy used for better reducing cardiovascular and death events in T2D patients, especially in those with high or very high cardiovascular risk, such as T2D patients with established atherosclerotic cardiovascular disease, heart failure, or chronic kidney disease.

This meta-analysis has three main limitations as follows. First, given the absence of specialized CVOTs comparing the cardiovascular/cerebrovascular outcomes between SGLT2i + GLP1RA combination therapy and SGLT2i/GLP1RA monotherapy, we could only conduct this meta-analysis by incorporating relevant observational studies and the studies reporting relevant subgroup/secondary/post hoc analyses of CVOTs (these subgroup analyses studies could be treated as observational studies since study groups of our interest did not derive from random allocation). Therefore, the findings of this meta-analysis are needed to be further validated by specialized CVOTs comparing the combination therapy with monotherapy. Second, since we included a limited number of studies and patients in this meta-analysis (especially, in some specific subgroups only one or two studies were included), the findings of this meta-analysis need to be confirmed by future more data. Last, due to the available data limited, in this meta-analysis we only evaluated the composite cardiovascular outcomes, such as MACE and the CVM/HHF composite, but failed to evaluate individual cardiovascular/cerebrovascular outcomes, such as separate myocardial infarction and stroke. Moreover, we failed to assess renal failure-associated endpoints, and failed to distinguish different SGLT2i and GLP1RA when comparing the combination therapy with monotherapy. Therefore, there is also a need for further studies to fill these knowledge gaps.

In summary, our meta-analysis identified that SGLT2i + GLP1RA combination therapy was significantly associated with reduced risks of MACE, CVM/HHF, and ACM compared with SGLT2i/GLP1RA monotherapy. These might support this combination therapy used for better reducing cardiovascular and death events in T2D patients, especially in those with high or very high cardiovascular risk.

## Abbreviations

CVOTs, cardiovascular outcome trials; SGLT2i, sodium-glucose cotransporter 2 inhibitors; GLP1RA, glucagon-like peptide-1 receptor agonists; T2D, type 2 diabetes; MACE, major adverse cardiovascular events; CVM, cardiovascular mortality; HHF, hospitalization for heart failure; ACM, all-cause mortality; HR, hazard ratio; CI, confidence interval.
