# Supplementary figures and images for: Meta-analysis assessing the effectiveness of SGLT2i+GLP1RA combination therapy versus monotherapy on cardiovascular and cerebrovascular outcomes in diabetic patients
Source: Front Physiol. 2022 Nov 7;13:1028486. doi: 10.3389/fphys.2022.1028486 (PMC9676496; doi:10.3389/fphys.2022.1028486)

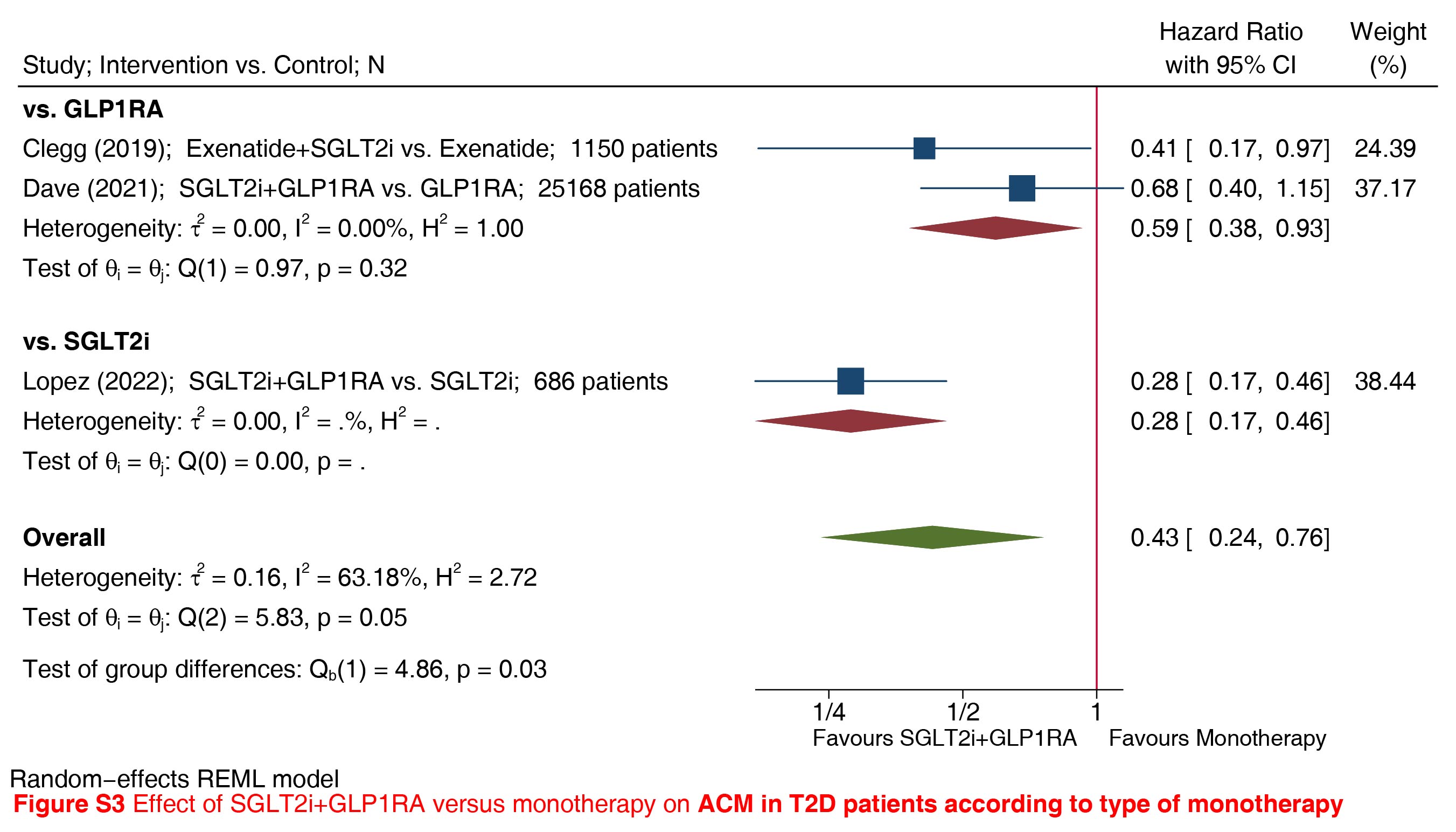

Supplement: Supplementary file 1 [file Image3.JPEG]

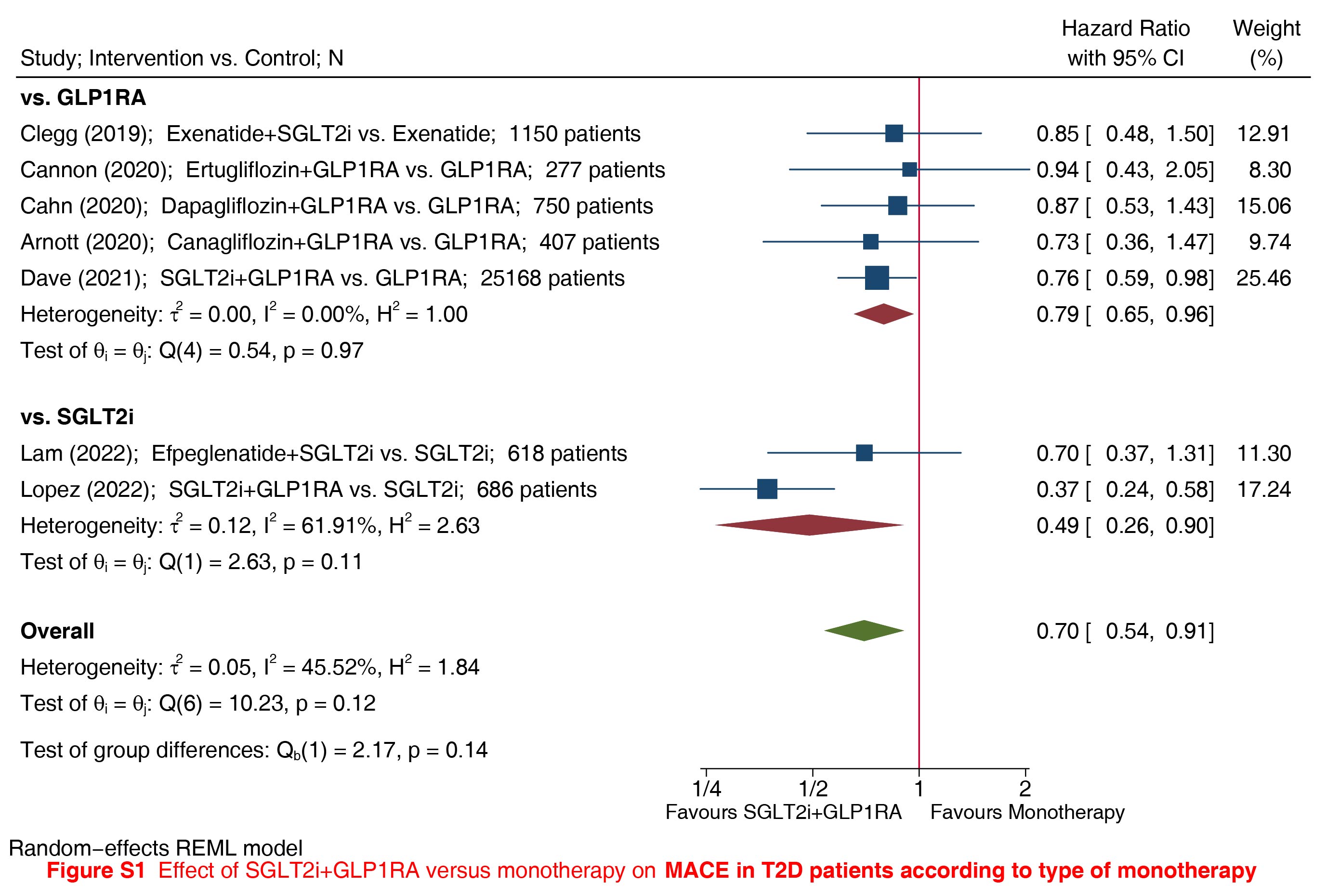

Supplement: Supplementary file 2 [file Image1.JPEG]

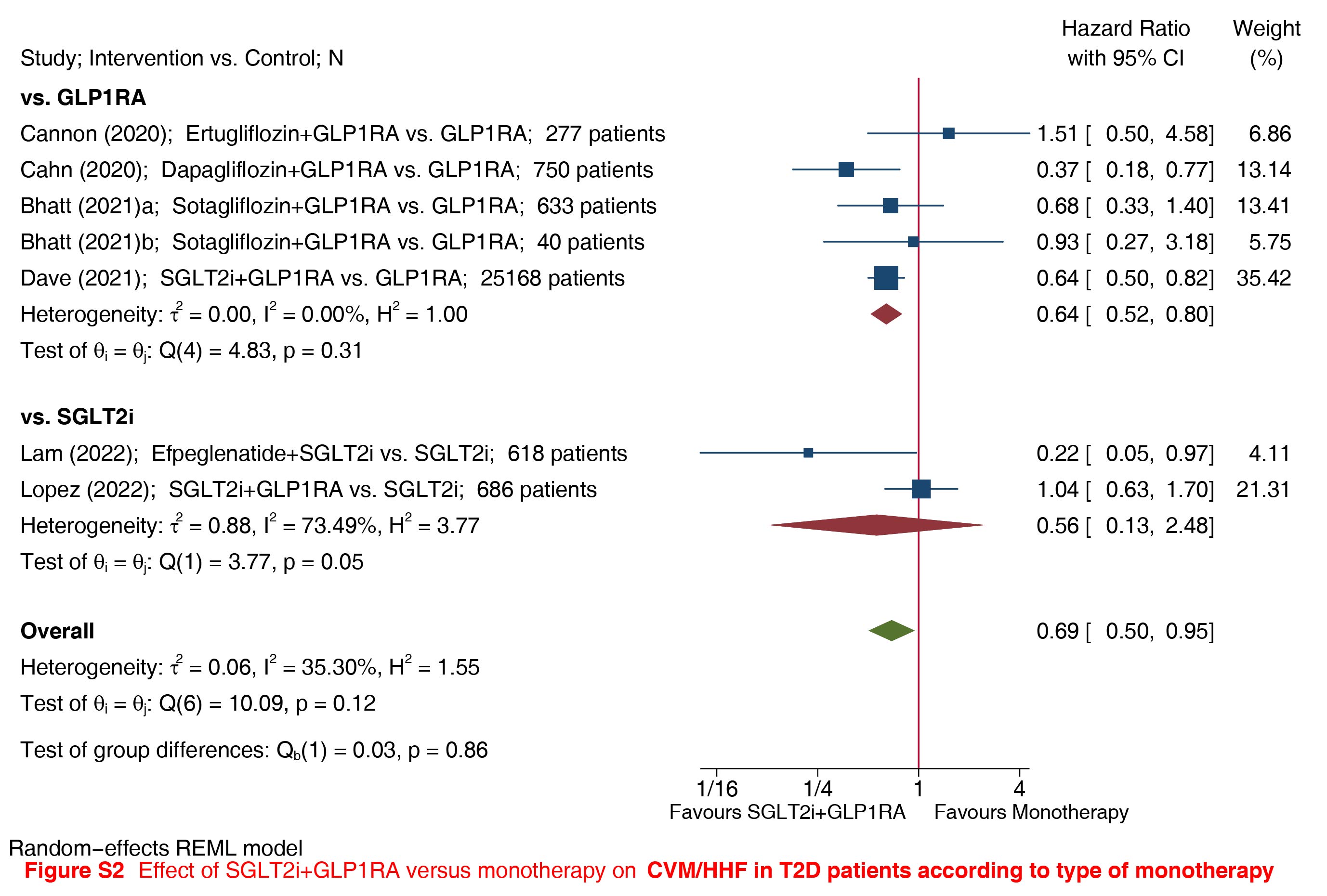

Supplement: Supplementary file 3 [file Image2.JPEG]
